# Draft Genome Sequence of Methicillin-Susceptible *Staphylococcus aureus* Strain 06BA18369, a Pathogen Associated with Skin and Soft Tissue Infections in Northern Saskatchewan, Canada

**DOI:** 10.1128/genomeA.00389-13

**Published:** 2013-06-27

**Authors:** Ryan R. McDonald, George R. Golding, James Irvine, Morag R. Graham, Shaun Tyler, Michael R. Mulvey, Paul N. Levett

**Affiliations:** Saskatchewan Disease Control Laboratory, Regina, Saskatchewan, Canadaa; Department of Biology, University of Regina, Regina, Saskatchewan, Canadab; National Microbiology Laboratory, Winnipeg, Manitoba, Canadac; Department of Medical Microbiology, University of Manitoba, Winnipeg, Manitoba, Canadad; Population Health Unit, Mamawetan Churchill River & Keewatin-Yatthé Health Regions & Athabasca Health Authority, LaRonge, Saskatchewan, Canadae; Department of Academic Family Medicine, University of Saskatchewan, Saskatoon, Saskatchewan, Canadaf

## Abstract

Here, we announce the draft sequence of a representative methicillin-susceptible *Staphylococcus aureus* (MSSA) isolate (06BA18369) whose strain type (*spa* type t311) was commonly isolated from skin and soft tissue coinfections with *Streptococcus pyogenes*. This strain sequence provides insight into a highly successful community-associated MSSA strain type.

## GENOME ANNOUNCEMENT

Efforts to elucidate the molecular epidemiology of *Staphylococcus aureus* in Canada have been largely focused on methicillin-resistant *S. aureus* (MRSA) infections acquired in health care settings and the emergence of MRSA that is associated with at-risk community populations (1–5). However, strains of methicillin-susceptible *S. aureus* (MSSA) isolated from community-acquired infections are seldom the subject of surveillance for antimicrobial susceptibility and strain typing. In northern Saskatchewan communities, MSSA was found to cause a high rate of skin and soft tissue infections (SSTI), in addition to those caused by community-acquired MRSA (CA-MRSA) USA400 strains (6). While MSSA isolates exhibited a greater genetic diversity than the collection of MRSA isolates, many of these MSSA isolates were coisolated with *Streptococcus pyogenes* in SSTI; MSSA *spa* type t311 and *S. pyogenes emm* type 41.2 made up the most commonly encountered pair of bacterial subtypes (7).

*S. aureus* 06BA18369 (*spa* type t311, sequence type 5 [ST5]) was isolated from an SSTI specimen, which also harbors *S. pyogenes* 06BA18369 (*emm* type 41.2, ST579), and was chosen for further genomic investigation.

Genome sequence data from *S. aureus* 06BA18369 were generated by the 454 Life Sciences GS-FLX genome sequencer (Roche Applied Science, Laval, Quebec, Canada) and consist of 262,689 reads with an average read length of 246 bases. Pyrosequencing reads were assembled with Newbler v1.1.03.24 (Roche) to generate 79 contigs of >500 bp. A fosmid genomic DNA library was screened to identify cloned fragments that were useful for gap closure. Direct sequencing reads of cloned fragments spanning gaps were assembled with existing contigs using Gap4 of the Staden package (8), and resulted in 59 contigs totaling 2,865,757 bases, with a G+C content of 32.7%.

Initial sequence analysis using GeneMark (9) revealed the presence of 2,793 putative protein-coding genes. tRNAscan (10) identified 59 tRNA genes, while RNAmmer (11) identified 6 rRNA regions.

This draft genome contains the genomic islands νSaα and νSaβ, with the latter carrying the cytotoxin genes *lukD* and *lukE*, as well as enterotoxin genes *seg*, *sen*, *sei*, *sem*, and *seo*, and the truncated genes ψ*ent1* and ψ*ent2*. In addition, *S. aureus* pathogenicity island 2 (SaPI2) is present, harboring the superantigen-encoding genes *sec*, *sel*, and *tst*. Four putative prophage sequences were identified using PHAST (12) as belonging to the bacteriophage families φSa1, φSa3, φSa6, and φSa7 (13). φSa3 harbors the staphylokinase and staphylococcal complement inhibitor-encoding genes *sak* and *scin*, respectively.

*S. aureus* 06BA18369 contains a staphylococcal cassette chromosome (SCC) element with a type 1 *ccr* gene complex, but it lacks a *mec* complex (14). This SCC (SCC_18369_) is approximately 18.5 kb in size and contains 16 open reading frames (ORFs). BLASTn searches of ORFs returned sequences homologous to the SCC regions originating from *S. aureus* strain MSSA476 (15), *Staphylococcus hominis* GIFU12263 (16), and *Staphylococcus epidermidis* isolates ATCC 12228 (17) and VCU120. Most notably, SCC_18369_ harbors an ORF with 100% nucleotide identity to the putative fusidic acid resistance gene *far1* harbored by SCC_476_ of *S. aureus* MSSA476 (15).

Further work is required to ascertain the prevalence of MSSA harboring SCC in individuals in northern Saskatchewan communities.

### Nucleotide sequence accession numbers.

This Whole-Genome Shotgun project has been deposited at GenBank under the accession no. ARXY00000000. The version described in this paper is the first version, accession no. ARXY01000000.

## References

[1] Simor A Boyd D Louie L McGeer A Mulvey M Willey B the Canadian Nosocomial Infection Surveillance Program 1999 Characterization and proposed nomenclature of epidemic strains of MRSA in Canada. Can. J. Infect. Dis. 10:333–336 22346391 10.1155/1999/769601 PMC3250711

[2] Simor AE Ofner-Agostini M Bryce E McGeer A Paton S Mulvey MR, Canadian Hospital Epidemiology Committee and Canadian Nosocomial Infection Surveillance Program, Health Canada 2002 Laboratory characterization of methicillin-resistant *Staphylococcus aureus* in Canadian hospitals: results of 5 years of national surveillance, 1995–1999. J. Infect. Dis. 186:652–660 12195352 10.1086/342292

[3] Mulvey MR MacDougall L Cholin B Horsman G Fidyk M Woods S Saskatchewan 2005 Community-associated methicillin-resistant *Staphylococcus aureus*, Canada. Emerg. Infect. Dis. 11:844–850 15963278 10.3201/eid1106.041146 PMC3367573

[4] Gilbert M MacDonald J Gregson D Siushansian J Zhang K Elsayed S Laupland K Louie T Hope K Mulvey M Gillespie J Nielsen D Wheeler V Louie M Honish A Keays G Conly J 2006 Outbreak in Alberta of community-acquired (USA300) methicillin-resistant *Staphylococcus aureus* in people with a history of drug use, homelessness or incarceration. CMAJ 175:149–154 16804118 10.1503/cmaj.051565 PMC1490001

[5] Nichol KA Adam HJ Hussain Z Mulvey MR McCracken M Mataseje LF Thompson K Kost S Lagacé-Wiens PR Hoban DJ Zhanel GG, Canadian Antimicrobial Resistance Alliance (CARA) 2011 Comparison of community-associated and health care-associated methicillin-resistant *Staphylococcus aureus* in Canada: results of the CANWARD 2007–2009 study. Diagn. Microbiol. Infect. Dis. 69:320–325 21353960 10.1016/j.diagmicrobio.2010.10.028

[6] Golding GR Levett PN McDonald RR Irvine J Quinn B Nsungu M Woods S Khan M Ofner-Agostini M Mulvey MR, Northern Antibiotic Resistance Partnership 2011 High rates of *Staphylococcus aureus* USA400 infection, northern Canada. Emerg. Infect. Dis. 17:722–725 21470471 10.3201/eid1704.100482 PMC3377391

[7] McDonald RR Golding GR Levett PN Yost CK Irvine J Horsman GB Mulvey MR, the Northern Antibiotic Resistance Partnership 2008 Characterization of *Streptococcus pyogenes* and *Staphylococcus aureus* isolates associated with mixed skin and soft tissue infections in northern Saskatchewan. Can. J. Infect. Dis. Med. Microbiol. 19:77–142

[8] Bonfield JK Smith KF Staden R 1995 A new DNA sequence assembly program. Nucleic Acids Res. 23:4992–4999 8559656 10.1093/nar/23.24.4992 PMC307504

[9] Besemer J Borodovsky M 2005 GeneMark: web software for gene finding in prokaryotes, eukaryotes and viruses. Nucleic Acids Res. 33:W451–W454 15980510 10.1093/nar/gki487 PMC1160247

[10] Schattner P Brooks AN Lowe TM 2005 The tRNAscan-SE, snoscan and snoGPS web servers for the detection of tRNAs and snoRNAs. Nucleic Acids Res. 33:W686–W689 15980563 10.1093/nar/gki366 PMC1160127

[11] Lagesen K Hallin P Rødland EA Staerfeldt HH Rognes T Ussery DW 2007 RNAmmer: consistent and rapid annotation of ribosomal RNA genes. Nucleic Acids Res. 35:3100–3108 17452365 10.1093/nar/gkm160 PMC1888812

[12] Zhou Y Liang Y Lynch KH Dennis JJ Wishart DS 2011 PHAST: a fast phage search tool. Nucleic Acids Res. 39:W347–W352 21672955 10.1093/nar/gkr485 PMC3125810

[13] Lindsay JA Holden MT 2004 Staphylococcus aureus: superbug, super genome? Trends Microbiol. 12:378–385 15276614 10.1016/j.tim.2004.06.004

[14] International Working Group on the Classification of Staphylococcal Cassette Chromosome Elements (IWG-SCC). 2009 Classification of staphylococcal cassette chromosome *mec* (SCC*mec*): guidelines for reporting novel SCC*mec* elements. Antimicrob. Agents Chemother. 53:4961–4967 19721075 10.1128/AAC.00579-09 PMC2786320

[15] Holden MT Feil EJ Lindsay JA Peacock SJ Day NP Enright MC Foster TJ Moore CE Hurst L Atkin R Barron A Bason N Bentley SD Chillingworth C Chillingworth T Churcher C Clark L Corton C Cronin A Doggett J Dowd L Feltwell T Hance Z Harris B Hauser H Holroyd S Jagels K James KD Lennard N Line A Mayes R Moule S Mungall K Ormond D Quail MA Rabbinowitsch E Rutherford K Sanders M Sharp S Simmonds M Stevens K Whitehead S Barrell BG Spratt BG Parkhill J 2004 Complete genomes of two clinical *Staphylococcus aureus* strains: evidence for the rapid evolution of virulence and drug resistance. Proc. Natl. Acad. Sci. U. S. A. 101:9786–9791 15213324 10.1073/pnas.0402521101 PMC470752

[16] Katayama Y Takeuchi F Ito T Ma XX Ui-Mizutani Y Kobayashi I Hiramatsu K 2003 Identification in methicillin-susceptible *Staphylococcus hominis* of an active primordial mobile genetic element for the staphylococcal cassette chromosome *mec* of methicillin-resistant *Staphylococcus aureus*. J. Bacteriol. 185:2711–2722 12700250 10.1128/JB.185.9.2711-2722.2003 PMC154413

[17] Mongkolrattanothai K Boyle S Murphy TV Daum RS 2004 Novel non-*mecA*-containing staphylococcal chromosomal cassette composite island containing *pbp4* and *tagF* genes in a commensal staphylococcal species: a possible reservoir for antibiotic resistance islands in *Staphylococcus aureus*. Antimicrob. Agents Chemother. 48:1823–1836 15105141 10.1128/AAC.48.5.1823-1836.2004 PMC400556

